# Evaluation of a high-EPA oil from transgenic *Camelina sativa* in feeds for Atlantic salmon (*Salmo salar* L.): Effects on tissue fatty acid composition, histology and gene expression

**DOI:** 10.1016/j.aquaculture.2015.03.020

**Published:** 2015-07-01

**Authors:** M.B. Betancor, M. Sprague, O. Sayanova, S. Usher, P.J. Campbell, J.A. Napier, M.J. Caballero, D.R. Tocher

**Affiliations:** a Institute of Aquaculture, School of Natural Sciences, University of Stirling, Stirling FK9 4LA, United Kingdom; b Department of Biological Chemistry and Crop Protection, Rothamsted Research, Harpenden AL5 2JQ, United Kingdom; c Biomar Ltd., North Shore Road, Grangemouth FK3 8UL, United Kingdom; d Aquaculture Research Group, University of Las Palmas de Gran Canaria & ICCM, Instituto Universitario de Sanidad Animal, Trasmontaña s/n, 35413, Arucas, Las Palmas, Canary Islands, Spain

**Keywords:** EPA, Camelina, Aquaculture, Fish oil, Pyloric caeca microarray

## Abstract

Currently, one alternative for dietary fish oil (FO) in aquafeeds is vegetable oils (VO) that are devoid of omega-3 (n-3) long-chain polyunsaturated fatty acids (LC-PUFAs). Entirely new sources of n-3 LC-PUFA such as eicosapentaenoic (EPA) and docosahexaenoic (DHA) acids through de novo production are a potential solution to fill the gap between supply and demand of these important nutrients. *Camelina sativa* was metabolically engineered to produce a seed oil (ECO) with > 20% EPA and its potential to substitute for FO in Atlantic salmon feeds was tested. Fish were fed with one of the three experimental diets containing FO, wild-type camelina oil (WCO) or ECO as the sole lipid sources for 7 weeks. Inclusion of ECO did not affect any of the performance parameters studied and enhanced apparent digestibility of individual n-6 and n-3 PUFA compared to dietary WCO. High levels of EPA were maintained in brain, liver and intestine (pyloric caeca), and levels of DPA and DHA were increased in liver and intestine of fish fed ECO compared to fish fed WCO likely due to increased LC-PUFA biosynthesis based on up-regulation of the genes. Fish fed ECO showed slight lipid accumulation within hepatocytes similar to that with WCO, although not significantly different to fish fed FO. The regulation of a small number of genes could be attributed to the specific effect of ECO (311 features) with metabolism being the most affected category. The EPA oil from transgenic Camelina (ECO) could be used as a substitute for FO, however it is a hybrid oil containing both FO (EPA) and VO (18:2n-6) fatty acid signatures that resulted in similarly mixed metabolic and physiological responses.

## Highlights

• A high-EPA oil (ECO) derived from transgenic Camelina was evaluated as a substitute for fish oil (FO) in salmon feeds
• Substitution of FO by ECO did not affect fish performance and enhanced apparent digestibility of fatty acids compared to FO
• ECO-fed fish maintained high levels of EPA in their tissues and showed no major transcriptomic changes in pyloric caeca.
• ECO was demonstrated to be a potentially suitable substitute for FO in salmon feeds.

## 1 Introduction

World population is growing at a rate of 1.4% per year, which means that by 2050 there will be 9.1 billion people in the world (FAO, 2009). This translates into greatly increased requirements for animal protein for human consumption. Regarding seafood, this must be farmed, as there is no expectation of increased production from capture fisheries (FAO, 2014). Traditionally fish meal and fish oil (FO) have been the dominant raw materials for aquaculture feeds, but this is not sustainable as these are limited and finite resources and supply cannot meet future demand. Therefore, it is necessary to find more sustainable alternatives to these marine ingredients. Vegetable oils (VO) are the principle candidates as substitutes for FO in fish feeds and many studies have demonstrated the feasibility of their use (Turchini et al., 2011). However, VO are rich in C_18_ polyunsaturated fatty acids (PUFA) such as linoleic (LA, 18:2n-6) and α-linolenic (ALA; 18:3n-3) acids but lack the omega-3 (n-3) long-chain PUFA (LC-PUFA), eicosapentaenoic (EPA; 20:5n-3) and docosahexaenoic (DHA; 22:6n-3) acids. Dietary intake of n-3 LC-PUFA has a range of well-established beneficial effects in human health, mitigating pathological conditions such as cardiovascular and inflammatory diseases, and some cancers, and is important for neural development (Campoy et al., 2012; Delgado-Lista et al., 2012; Gil et al., 2012; Laviano et al., 2013; Miles and Calder, 2012; Raatz et al., 2013; Rangel-Huerta et al., 2012). Generally, replacement of dietary FO with VO has resulted in a lower level of n-3 LC-PUFA in the flesh of farmed fish, reducing their nutritional value to human consumers (Tocher, 2003; Turchini et al., 2011). Consequently, the problem in replacing FO is not finding an alternative lipid energy source, as VO satisfy this well, but finding a source of n-3 LC-PUFA.

The n-3 LC-PUFA are produced in marine microalgae (Harwood and Guschina, 2009) and are passed up the food chain, accumulating in fish lipids (Tocher, 2003). However, culture of microalgae themselves as a source of n-3 LC-PUFA has significant biological and technological problems that contribute to low supply and high production costs, currently and severely limiting their potential commercial use (Tocher, in press). Indeed, some predict that it may be impossible for microalgal oils to satisfy demand for n-3 LC-PUFA on a global scale (Turchini et al., 2010). However, one practical approach to an alternative source of n-3 LC-PUFA is to use microalgal genes for the metabolic engineering of oilseed crops with the capacity to synthesise EPA and DHA (Haslam et al., 2013; Sayanova and Napier, 2011). The modest running costs in conjunction with the scalability of agriculture-based production systems highlight the potential of transgenic plants as “green factories” (Dyer et al., 2008). Among the possible oilseeds, *Camelina sativa*, a member of the Brassicaceae family, is an attractive crop platform for such metabolic engineering, based on its low input cost and ease of transformation (Nguyen et al., 2013; Ruiz-Lopez et al., 2014). In addition, wild-type camelina is naturally rich in ALA (~ 45%) (Gunstone et al., 2007), the substrate fatty acid for the biosynthesis of n-3 LC-PUFA.

The transgenic *C. sativa* tested in the present study contained a suite of five microalgal genes to produce EPA de novo in the seeds (Ruiz-Lopez et al., 2014). Lipid content in seeds was approximately 45%, with EPA accounting for over 20% of total fatty acids in the seed oil (ECO) (Ruiz-Lopez et al., 2014). The ALA content in the metabolically transformed seeds was reduced in contrast to the oil from wild-type camelina (WCO) as a result of conversion to EPA (Ruiz-Lopez et al., 2014). Recently, we evaluated ECO in feeds for Atlantic salmon (*Salmo salar* L.) post-smolt, showing that fish performance and flesh nutritional quality for the human consumer in terms of total n-3 LC-PUFA were similar in fish fed ECO and fish fed FO (Betancor et al., 2015). Furthermore, lipid transcriptomic analysis indicated that the EPA:DHA ratio had a greater influence on hepatic gene expression than the absolute dietary level of EPA (Betancor et al., 2015).

The overall objective of the present study was to determine the effects of ECO as a replacement for dietary FO in feeds for Atlantic salmon with a focus on intestinal function. Triplicate groups of Atlantic salmon post-smolts were fed diets containing either FO, WCO or ECO as the sole lipid source for 7 weeks. Specific analyses included apparent digestibility, fatty acid composition, gene expression and histology of intestinal tissues. In addition, results prompted further histological analysis of liver and head kidney, and brain fatty acid composition was determined to assess the extent of DHA production from dietary EPA in neural tissue.

## 2 Materials and methods

### 2.1 Vector construction

A construct containing a cassette of five genes was used for transformation (Ruiz-Lopez et al., 2014). Briefly, the five-gene construct contained a set of genes optimised for EPA synthesis: a Δ6-desaturase gene from *Ostococcus tauri* (OtΔ6), a Δ6 fatty acid elongase gene from *Physcomitrella patens* (PSE1) a Δ5-desaturase gene from *Thraustochytrium* sp. (TcΔ5), a Δ12-desaturase gene from *Phytophthora sojae* (PsΔ12) and an ω3-desaturase from *Phytophthora infestans* (Pi- ω3) as described in detail previously (Ruiz-Lopez et al., 2014). All genes were individually cloned under the control of seed-specific promoters, and then combined into a single T-DNA transformation vector as described previously (Ruiz-Lopez et al., 2013). The destination vector contained an NPTII gene with the nos promoter as a selection marker. All open reading frames for desaturases and elongases were re-synthesised and codon-optimised for expression in *C. sativa*.

### 2.2 Generation of transgenic plants and production of oil

Transgenic *C. sativa lines* were generated essentially as described previously (Lu and Kang, 2008; Nguyen et al., 2013). Briefly, the vectors were transferred into *Agrobacterium tumefacians* strain AGL1 and *C. sativa* inflorescences immersed in the *Agrobacterium* suspension for 30 s without the application of vacuum. Visual screening for DsRed activity was used to select transgenic seeds expressing the EPA biosynthetic pathway. Seeds harvested from transformed plants were illuminated with green LED light and fluorescent seeds identified using a red lens filter. No phenotypic perturbation was observed as a result of modification of the seed oil composition. Full details are in Ruiz-Lopez et al. (2014).

*C. sativa* was grown in a controlled-environment glasshouse at 23 °C day/18 °C night, 50–60% humidity, and kept under a 16 h photoperiod (long day) at 250 lmol m-2 s-1. Oil was produced from seeds by cold-pressing and solvent extraction to maximise yield (PPM, Magdeburg, Germany). The anti-oxidant ethoxyquin (300 ppm) was added to stabilise the final product.

### 2.3 Diets and feeding trial

Three isonitrogenous and isoenergetic diets were formulated to satisfy the nutritional requirements of salmonid fish (NRC, 2011) (Table 1). The diets supplied 46 g.kg^− 1^ crude protein and 21 g.kg^− 1^ crude lipid at a crude energy level of 22.5 MJ.kg^− 1^ and were manufactured at BioMar Tech-Centre (Brande, Denmark). The three feeds were produced by vacuum coating identical dry basal extruded pellets with either fish oil (FO), wild-type Camelina oil (WCO) or EPA–Camelina oil (ECO) and were named according to the oils used. Non-defatted fishmeal was employed as the major protein source to ensure EFA requirements were met (NRC, 2011). Yttrium oxide was added to the experimental diets (0.5 g kg^− 1^) as an inert marker for calculation of lipid and fatty acid digestibility.

A total of 405 post-smolt Atlantic salmons with an average body weight of 82.5 ± 8.1 g (mean ± S.D.) were distributed into 9 seawater tanks (45 per tank) and fed one of the three experimental feeds in triplicate for 7 weeks. Prior to the start of the experimental period, during a 1 week acclimation period, fish were feeding the WCO diet. The experimental system comprised 1 m^2^, 500 L tanks supplied by flow-through seawater (15 L min^− 1^) at ambient temperature that averaged 10.2 ± 0.6 °C. Experimental feeds were delivered in excess by automatic disc feeders with an automated uneaten feed collection system in order to determine accurate feed efficiency.

### 2.4 Sample collection and digestibility

At the end of the trial, fish were not fed for 48 h prior to being anaesthetised and killed by overdose with metacaine sulphonate (MS222). Nine fish per tank were used for biometric measurements (hepato-somatic and viscera-somatic indices) and tissue analyses. Samples of anterior intestine, pyloric caeca and brain from 3 fish per tank were immediately frozen in liquid nitrogen and stored at − 70 °C prior to total lipid and fatty acid analyses. Further samples of pyloric caeca were collected from six fish per treatment (two per tank) and stabilised in RNAlater® (Sigma, Poole, UK) prior to RNA extraction.

After 7 weeks of feeding, samples of salmon faeces were collected from 12 fish randomly selected from each tank and the faecal samples pooled by tank. Fish were anesthetised with MS222 and faecal samples collected from the hind gut region by gently squeezing the ventral abdominal area (Austreng, 1978). Faecal samples were stored − 20 °C prior to lipid and fatty acid analysis. The apparent digestibility coefficient (ADC) of lipid and selected fatty acids was calculated as: 100 − [100 × (Y_2_O_3_ concentration in feed / Y_2_O_3_ concentration in faeces) × (lipid or fatty acid concentration in faeces / lipid or fatty acid concentration in feed)]. The concentration of individual fatty acids in diets and faeces were calculated based on the relative proportion of each fatty acid compared with a known amount of the internal standard (17:0) added and the total lipid content determined in the samples.

### 2.5 Tissue lipid content and fatty acid composition

Samples of anterior intestine, pyloric caeca and brain from three fish per tank were prepared as pooled homogenates (n = 3 per treatment) and total lipid extracted from 1 g by homogenising in chloroform/methanol (2:1, v/v) using an Ultra-Turrax tissue disrupter (Fisher Scientific, Loughborough, UK), and content determined gravimetrically (Folch et al., 1957). Fatty acid methyl esters (FAMEs) were prepared from total lipid by acid-catalysed transesterification at 50 °C for 16 h (Christie, 2003), and FAMEs were extracted and purified as described previously (Tocher and Harvie, 1988). FAMEs were separated and quantified by gas–liquid chromatography using a Fisons GC-8160 (Thermo Scientific, Milan, Italy) equipped with a 30 m × 0.32 mm i.d. × 0.25 μm ZB-wax column (Phenomenex, Cheshire, UK), on-column injector and a flame ionisation detector. Data were collected and processed using Chromcard for Windows (version 2.01; Thermoquest Italia S.p.A., Milan, Italy). Individual FAME was identified by comparison to known standards (Supelco™ 37-FAME mix; Sigma-Aldrich Ltd., Poole, UK) and published data (Tocher and Harvie, 1988).

### 2.6 Histology analysis

Samples of the intestine, liver and head kidney from 2 fish per tank (n = 6 per treatment) were fixed in 4% buffered formalin dehydrated through graded alcohol, then xylene, and finally embedded in paraffin wax. The paraffin blocks were sectioned at 3 μm and stained with haematoxylin and eosin (Martoja and Martoja-Pearson, 1970) before blind examination under a light microscope. Stained sections of liver were assessed for cytoplasmic lipid vacuolization using a four graded examination scheme: 0, not observed; 1, few; 2, medium; 3, and severe. Posterior intestine sections were examined for integrity of the intestinal mucosa and the presence of any inflammatory response. The surface corresponding to melanomacrophage centres in the head kidney was visualised and quantified using a computerised image analysis package (Image-Pro Plus®, Media Cybernetics, Maryland, USA). Five areas of each head kidney section were quantified. By selecting ranges of pixel values in colour images the pixels associated with black could be distinguished. The number of selected pixels was then quantified using a particle analysis operation and by counting the area of all bright objects (in pixels).

### 2.7 RNA extraction

Pyloric caeca from six individual fish per dietary treatment were homogenised in 1 mL of TriReagent® (Sigma-Aldrich, Dorset, UK) RNA extraction buffer using a bead tissue disruptor (Bio Spec, Bartlesville, Oklahoma, USA). Total RNA was isolated following manufacturer's instructions and quantity and quality determined by spectrophotometry using a Nanodrop ND-1000 (Labtech Int., East Sussex, UK) and electrophoresis using 500 ng of total RNA in a 1% agarose gel.

### 2.8 Microarray hybridizations and image analysis

Transcriptome analysis of pyloric caeca tissue was performed using an Atlantic salmon custom-made oligoarray with 44 k features per array in a four-array-per-slide format (Agilent Technologies UK Ltd., Wokingham, UK). The probes were co-designed by researchers at the Institute of Aquaculture (University of Stirling, UK) and the Norwegian Institute of Food, Fisheries and Aquaculture Research (Nofima, Tromsø, Norway). Microarray data are available in the ArrayExpress database under accession number E-MTAB-3268. A dual-label experimental design was employed for the microarray hybridisations with Cy3-labelled test samples competitively hybridised to a common Cy5-labelled pooled-reference per array. A total of 18 arrays were utilised, one array per individual fish. The common reference was a pool of equal amounts of amplified RNA from all test samples.

Indirect labelling methodology was employed in preparing the microarray targets. Amplified antisense RNA (aRNA) was produced from each RNA sample using TargetAmpTM 1-Round Aminoallyl-aRNA Amplification Kit 101 (Epicentre, Madison, Wisconsin, USA), as per manufacturer's methodology, followed by Cy3 or Cy5 fluor incorporation through a dye-coupling reaction. Microarray hybridisations were performed in SureHyb hybridisation chambers in a DNA Microarray Hybridisation Oven (Agilent Technologies). For each hybridisation, 825 ng of Cy3-labelled experimental biological replicate and Cy5-labelled reference pool were combined and total volume was made up to 35 μl with nuclease-free water. Detailed information regarding the microarray hybridisations and image analysis has been published previously (Morais et al., 2011a). The salmon custom array has been validated in many previous studies and for this particular study it had already been validated in liver tissue (Betancor et al., 2015).

### 2.9 Statistical analysis

All data are means ± S.E. (n = 3) unless otherwise specified. Percentage data were subjected to arcsin square-root transformation prior to statistical analyses. Data were tested for normality and homogeneity of variances with Levene's test prior to one-way analysis of variance followed by a Tukey–Kramer HSD multiple comparisons of means. All statistical analyses were performed using SPSS software (IBM SPSS Statistics 19; SPSS Inc., Chicago, IL, USA). Statistical analysis of microarray hybridisation data was performed in GeneSpring GX version 12.6.1 (Agilent Technologies, Wokingham, Berkshire, UK) using a Welch (unpaired unequal variance) t-test, at 0.05 significance. No multiple test correction was employed as previous analyses indicated that they were over-conservative for these nutritional data (Morais et al., 2011a,b). Data were submitted to the Kyoto Encyclopedia of Genes and Genomes (KEGG; Kanehisa and Goto, 2000) for biological function analysis. Gene expression results were analysed using the relative expression software tool (REST 2009; http://www.gene-quantification.info/), which employs a pairwise fixed reallocation randomization test (10,000 randomizations) with efficiency correction (Pfaffl et al., 2002) to determine the statistical significance of expression ratios (gene expression fold changes) between two treatments.

## 3 Results

### 3.1 Fish performance and digestibility

**Fig. 1:**
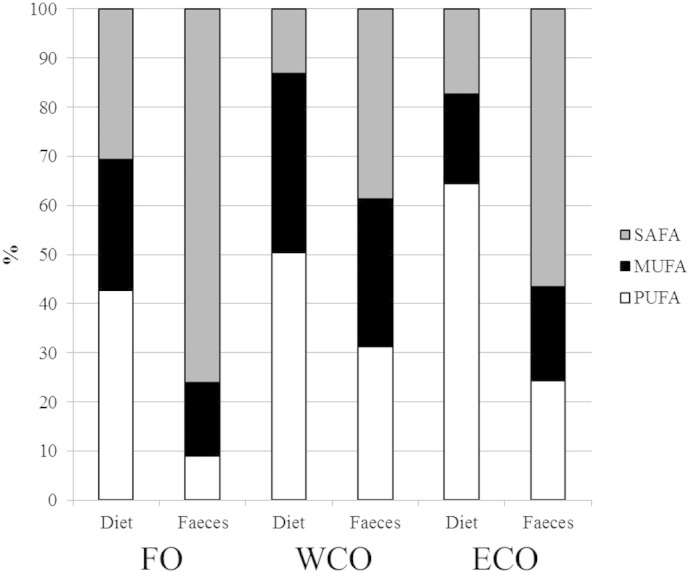
Fatty acid compositions of the three experimental feeds and faeces (area %) showing preferential order of absorption with differing degree of unsaturation of dietary fatty acids.

At the end of the trial, fish from all treatments doubled more than their previous weight and no significant mortality was recorded (Table 2). No differences were found in any of the performance/biometric parameters studied among the three dietary treatments. Apparent digestibility coefficients (ADC) were obtained for lipids and fatty acids using yttrium as an inert marker. Lipid digestibility was higher to fish fed ECO and WCO compared to fish fed FO, with no difference between the VO diets (Table 3). There was a trend showing higher saturated fatty acids (SAFA) in faeces relative to the feeds (Fig. 1), with the ADC for saturated fatty acids between 78.8 and 97.6%, generally slightly lower than the ADCs for the other fatty acids (Table 3). By contrast, PUFA were generally lower in the faeces compared to feeds with ADCs ranging from 92–> 99%, whereas monounsaturated fatty acids (MUFA) did not markedly vary between diet and faeces with ADCs from 95–> 99% (Fig. 1). There were no major differences in digestibility of most individual fatty acids between the ECO and WCO feeds, and digestibility of EPA was highest in the ECO feeds (99.6%; Table 3).

### 3.2 Lipid contents and fatty acid compositions of tissues

Lipid content in pyloric caeca was slightly lower, albeit non-significant, in fish fed the VO diets compared to fish fed FO (Table 4). Dietary treatment had no significant effect on lipid content in anterior intestine (Table 5) or in brain, which had the most consistent and stable lipid content among the dietary treatments (Table 6). The fatty acid compositions of the tissues showed some tissue-specific differences although they all generally largely reflected dietary fatty acid compositions. Therefore, pyloric caeca, anterior intestine and brain of fish fed the ECO diet had higher proportions of EPA than fish fed both FO and WCO diets (Tables 4–6). The percentages of docosapentaenoic acid (DPA; 22:5n-3) were significantly higher in all tissues of fish fed ECO compared to fish fed WCO, and the proportions of DHA were also consistently higher albeit not always significantly (Tables 4–6). In general terms, dietary effects on fatty acid composition were not as pronounced in brain as in intestinal tissues, with no differences among treatments in any of the totals for fatty acid groups. The proportions of DHA were also stable in brain, with its levels higher relative to dietary level especially for the ECO and WCO diets, reflecting its importance in nervous tissue (Table 6).

The fatty acid profiles of pyloric caeca and anterior intestine also reflected dietary fatty acid compositions although, in contrast to brain, differences were found in the fatty acid totals. Fatty acid profiles were similar in both intestinal tissues, with differences observed in DHA contents, with fish fed ECO showing intermediate values, higher than those observed in WCO-fed fish, but lower than the levels found in fish fed FO (Tables 4 and 5). The proportions of total n-6 PUFA were highest in pyloric caeca and anterior intestine of fish fed ECO, due to higher levels of 18:2n-6, 20:4n-6, 22:4n-6 and 22:5n-6 (Tables 4 and 6). The proportion of total n-3 PUFA was higher in pyloric caeca of fish fed the ECO diet compared to fish fed WCO, whereas no difference was found in anterior intestine. Dietary ECO significantly increased the total of EPA + DPA + DHA in anterior intestine to levels similar to those observed in FO-fed fish (Table 5), whereas in pyloric caeca these levels were similar in ECO and WCO-fed fish and lower than those in fish fed FO (Table 4). Conversely, diet had no effect on the levels of EPA + DPA + DHA in the brain (Table 6).

### 3.3 Histology

The intestinal tissues showed no signs of any abnormal histology. Specifically, no inflammatory infiltration was observed in proximal and distal intestine or pyloric caeca from fish fed any of the dietary treatments. There was reduced vacuolisation in the proximal sections, probably due to the 48 h of starvation period prior to sampling, with no differences among the dietary treatments. Similarly, there were no major changes in liver histology among fish fed the different dietary treatments. Fish fed the FO diet showed regular hepatocyte morphology with large centrally located nuclei with few cytoplasmic lipid vacuoles that did not alter hepatocyte size or shape. A higher degree of vacuolisation was observed in the hepatocytes of fish fed the WCO and ECO diets, although no obvious structural changes, such as inflammation, necrosis or perivascular cuffing were observed. Consequently, scoring of liver lipid vacuolisation was slightly higher in fish fed the VO diets, although the difference between fish fed FO and fish fed ECO was not significant (Table 7). No differences were observed in the area occupied by melanomacrophages centres in the head kidney tissue among the different dietary treatments (Table 7). These centres were relatively abundant in number but quite diffused and occupying only small areas. No signs of inflammation or any other alteration was found in the head kidney tissue.

### 3.4 Responses of pyloric caeca transcriptome

**Fig. 2:**
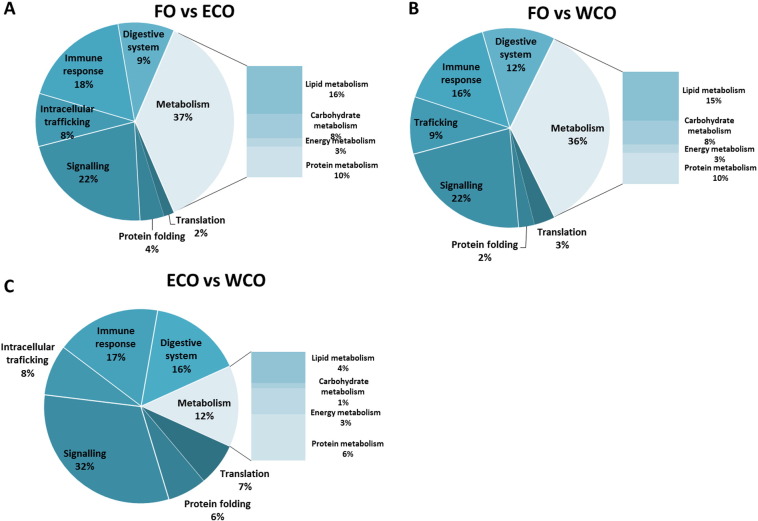
Distribution by categories of common differentially expressed genes in pyloric caeca between Atlantic salmon fed oil from transgenic camelina (ECO) (A) and wild-type camelina (WCO) (B) when compared to fish fed fish oil and between ECO and WCO (C) (Welch t-test, p < 0.05). Non-annotated genes and features corresponding to the same gene are not represented.

Statistical analysis of the microarray data returned a list of 2298 differentially expressed gene (DEG) features in the pyloric caeca between Atlantic salmon fed the ECO and FO diets, whereas 1152 DEG were found between fish fed the ECO and WCO diets (p < 0.05; Table 8). More genes were up-regulated than down-regulated in both comparisons with no difference between the percentages (59.5 and 57.4% up-regulated for ECO vs FO and ECO vs WCO, respectively). Most transcripts were regulated at a relatively low fold-change (FC) of < 1.5 for over 74% for both contrasts, with the highest percentage of higher FC (> 2.5) found among the down-regulated genes in the ECO vs WCO contrast (2.6%; Table 8). The DEG for each comparison were subjected to more detailed analysis by assigning KEGG orthology (KO) numbers and mapping them to a known compendium of metabolic pathways (KEGG). This analysis showed that both VO diets induced a similar transcriptomic response in the pyloric caeca in comparison to dietary FO. Thus, when the transcriptomes of pyloric caeca from Atlantic salmon fed both ECO and WCO were compared to that of fish fed the FO diet, the same cell processes were similarly affected, with the major categories being metabolism (37% for FO vs ECO and 36% for FO vs WCO), signalling (22% for both contrasts) and immune response (18% for FO vs ECO and 16% for FO vs WCO) (Fig. 2A and B). Within metabolism, the pathways most affected were that of lipid metabolism, accounting for 16 and 15% of the DEG in the FO vs ECO and FO vs WCO comparisons, respectively. By contrast, the main processes differentially regulated in pyloric caeca in the comparison between ECO and WCO were different, with signalling being the main category affected (32%) followed by immune response (17%) and digestive system (16%) (Fig. 2C). Features belonging to metabolic pathways only accounted for a 12% of the DEG and, within this, protein metabolism was more affected than lipid metabolism (6% vs 4%) (Fig. 2C).

**Fig. 3:**
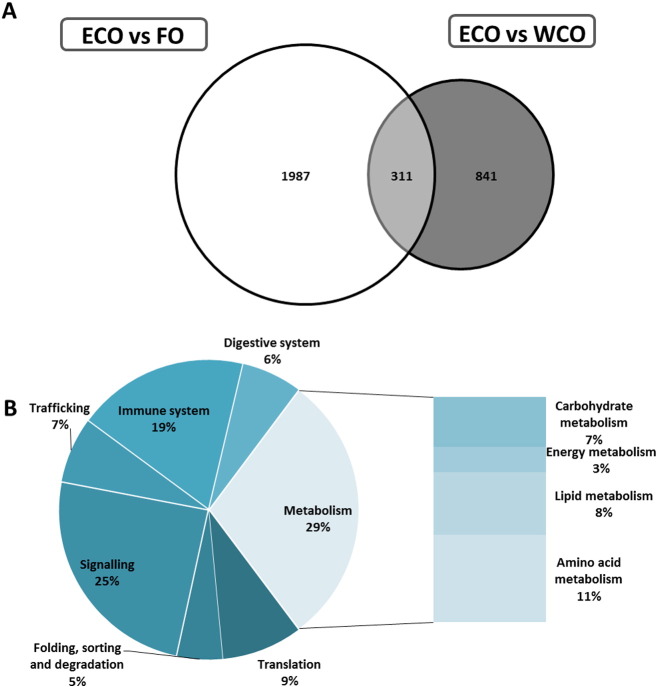
Impact of diet on pyloric caeca transcriptome of Atlantic salmon fed a diet containing oil from transgenic camelina (ECO) in comparison with fish fed diets containing fish oil (FO) or wild-type camelina oil (WCO). (A) Venn diagram representing the number of mRNA transcripts differentially expressed in the pyloric caeca of Atlantic salmon fed the ECO diet compared to fish fed the FO and WCO diets. The area of the circles is scaled to the number of transcripts (Welch test, p < 0.05). (B) Distribution by categories of common differentially expressed genes (311) in pyloric caeca between Atlantic salmon fed ECO compared to fish fed FO and WCO (Welch t-test, p < 0.05). Non-annotated genes and features corresponding to the same gene are not represented.

From all the DEG transcripts regulated, 1987 were exclusive to the ECO vs FO comparison, 841 to the ECO vs WCO and 311 were ECO-specific (common to both contrasts, p < 0.05; Fig. 3a). After removing non-annotated genes, KEGG analysis of the common 311 DEG returned 174 KO terms at p < 0.05 and revealed that the most affected biological categories were metabolism (29%), followed by signalling (25%) and immune system (19%). Within metabolism, amino acid metabolism was the main pathway affected (11%), followed by lipid metabolism (8%) (Fig. 3b). Analysis of the top 100 most significant common 311 DEG according to p value showed increased representation of translation (17.1%) and reduced representation of the metabolism category, mainly due to decreased amino acid metabolism features, whereas lipid metabolism remained well represented (Supplementary Table 1). The FC of these top 100 hits was generally < 1.5, and higher FCs were found for a few DEG; *large subunit ribosomal protein L6e*, *vesicle transport protein SEC22*, *activin receptor type – 1B*, *protein kinase, activation – induced cytidine deaminase*, *Zinc finger protein 576* and *tropomodulin 4*, all of which belonging to different functional categories. Among the top 100 most significant common genes, only *aspartate aminotransferase* showed expression changes in opposite directions, being up-regulated in ECO vs WCO and down-regulated in the ECO vs FO contrast (Supplementary Table 1).

From all the DEG transcripts regulated, 1987 were exclusive to the ECO vs FO comparison, 841 to the ECO vs WCO and 311 were ECO-specific (common to both contrasts, p < 0.05; Fig. 3A). After removing non-annotated genes, KEGG analysis of the common 311 DEG returned 174 KO terms at p < 0.05 and revealed that the most affected biological categories were metabolism (29%), followed by signalling (25%) and immune system (19%). Within metabolism, amino acid metabolism was the main pathway affected (11%), followed by lipid metabolism (8%) (Fig. 3B). Analysis of the top 100 most significant common 311 DEG according to p value showed increased representation of translation (17.1%) and reduced representation of the metabolism category, mainly due to decreased amino acid metabolism features, whereas lipid metabolism remained well represented (Supplementary Table 1). The FC of these top 100 hits was generally < 1.5, and higher FCs were found for a few DEG; *large subunit ribosomal protein L6e*, *vesicle transport protein SEC22*, *activin receptor type – 1B*, *protein kinase, activation – induced cytidine deaminase*, *Zinc finger protein 576* and *tropomodulin 4*, all of which belonging to different functional categories. Among the top 100 most significant common genes, only *aspartate aminotransferase* showed expression changes in opposite directions, being up-regulated in ECO vs WCO and down-regulated in the ECO vs FO contrast (Supplementary Table 1).

**Fig. 4:**
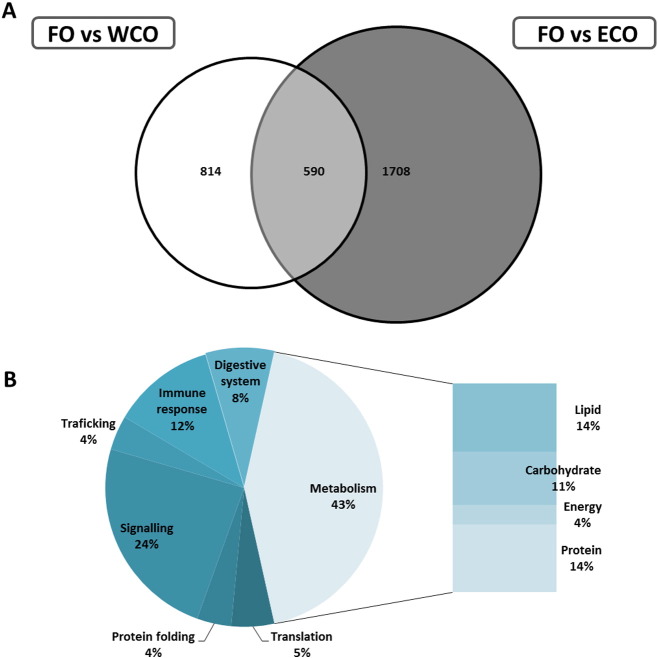
Impact of diet on pyloric caeca transcriptome of Atlantic salmon fed a diet containing fish oil (FO) in comparison with fish fed diets containing wild-type camelina oil (WCO) or oil from transgenic camelina (ECO). (A) Venn diagram representing the number of mRNA transcripts differentially expressed in the pyloric caeca of Atlantic salmon fed the FO diet compared to fish fed the WCO and ECO diets. The area of the circles is scaled to the number of transcripts (Welch test, p < 0.05). (B) Distribution by categories of common differentially expressed genes (311) in pyloric caeca between Atlantic salmon fed FO compared to fish fed WCO and ECO (Welch t-test, p < 0.05). Non-annotated genes and features corresponding to the same gene are not represented.

**Fig. 5:**
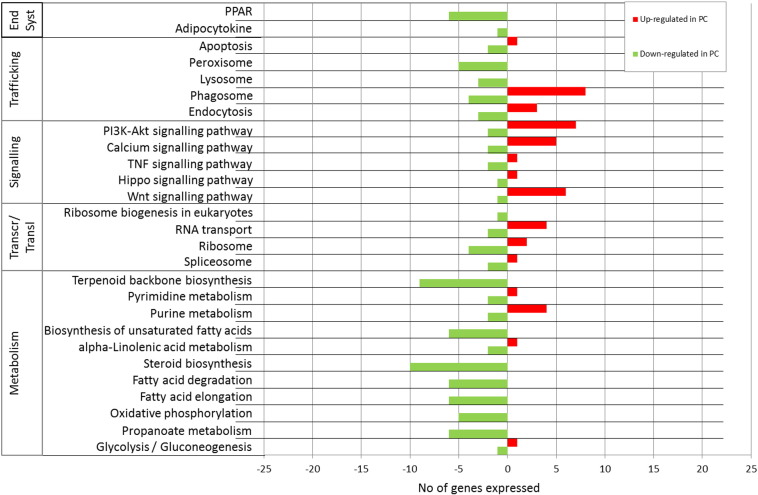
Ranking of differentially expressed pathways in Atlantic salmon pyloric caeca (PC) of common differentially expressed genes between fish fed wild type camelina oil (WCO) or high-EPA camelina oil (ECO) compared to fish fed fish oil (FO; FO/ECO and FO/WCO) diets for 7 weeks. Pathway analysis was performed using the Kyoto Encyclopedia of Genes and Genome (KEGG).

When the transcriptome of pyloric caeca of fish fed FO was compared with that of fish fed WCO, significant changes were observed in 1404 probes (p < 0.05; Table 8). Among these, 550 were commonly regulated in the FO vs ECO comparison (Fig. 4a). KEGG analysis of these FO-specific DEG showed that the main category regulated was that of metabolism, accounting for 43%, followed by signalling and immune response (24 and 12%, respectively; Fig. 4b). In the metabolism category, lipid and protein metabolism were equally represented (14%), followed by carbohydrate (11%) and energy (4%). Deeper analysis of the specific pathways showed, pathways with multiple regulated features including sterol/steroid biosynthesis with 10 genes down-regulated in fish fed FO (Fig. 5). Similarly, multiple down-regulated genes were also present in pathways of biosynthesis of unsaturated fatty acids (such as *delta-6 fatty acyl desaturase* (*fads2d6*) and *fatty acyl elongase 2* (*elovl2*)), fatty acid elongation and degradation (*long-chain fatty acid CoA lyase*; *acsbg*), and terpenoid backbone biosynthesis (*isopentenyl-diphosphate delta-isomerase*; *ipi* and *farnesyl diphosphate synthase*; *fdps*). Propanoate metabolism, a pathway of carbohydrate metabolism was also highly represented (6 probes, all down-regulated). The top 100 annotated DEG with a KO number, according to p value, showed that metabolism remained the main category affected, although lipid and protein metabolism were not equally represented (Supplementary Table 2). Within lipid metabolism, there was high representation of biosynthesis of unsaturated fatty acids (*delta-6-desaturase and elongation of very long chain fatty acids protein 2*), steroid biosynthesis (*delta-14-sterol reductase*, *delta-14-demethylase*, *cholesterol 7 alpha-monoxygenase* and *sterol-14-demethylase*) and terpenoid backbone biosynthesis (*farnesyl diphosphate synthase*, *squalene monooxygenase* and *isopentenyl-diphosphate delta-isomerase*). Immune system representation was reduced in the top 100 list (6.6%) with multiple features for the same gene, such as *T-cell receptor beta chain V region* and *major histocompatibility complex class II* (Supplementary Table 2).

When the transcriptome of pyloric caeca of fish fed FO was compared with that of fish fed WCO, significant changes were observed in 1404 probes (p < 0.05; Table 8). Among these, 550 were commonly regulated in the FO vs ECO comparison (Fig. 4A). KEGG analysis of these FO-specific DEG showed that the main category regulated was that of metabolism, accounting for 43%, followed by signalling and immune response (24 and 12%, respectively; Fig. 4B). In the metabolism category, lipid and protein metabolism were equally represented (14%), followed by carbohydrate (11%) and energy (4%). Deeper analysis of the specific pathways showed, pathways with multiple regulated features including sterol/steroid biosynthesis with 10 genes down-regulated in fish fed FO (Fig. 5). Similarly, multiple down-regulated genes were also present in pathways of biosynthesis of unsaturated fatty acids (such as *delta-6 fatty acyl desaturase* (*fads2d6*) and *fatty acyl elongase 2* (*elovl2*)), fatty acid elongation and degradation (*long-chain fatty acid CoA lyase*; *acsbg*), and terpenoid backbone biosynthesis (*isopentenyl-diphosphate delta-isomerase*; *ipi* and *farnesyl diphosphate synthase*; *fdps*). Propanoate metabolism, a pathway of carbohydrate metabolism was also highly represented (6 probes, all down-regulated). The top 100 annotated DEG with a KO number, according to p value, showed that metabolism remained the main category affected, although lipid and protein metabolism were not equally represented (Supplementary Table 2). Within lipid metabolism, there was high representation of biosynthesis of unsaturated fatty acids (*delta-6-desaturase and elongation of very long chain fatty acids protein 2*), steroid biosynthesis (*delta-14-sterol reductase*, *delta-14-demethylase*, *cholesterol 7 alpha-monoxygenase* and *sterol-14-demethylase*) and terpenoid backbone biosynthesis (*farnesyl diphosphate synthase*, *squalene monooxygenase* and *isopentenyl-diphosphate delta-isomerase*). Immune system representation was reduced in the top 100 list (6.6%) with multiple features for the same gene, such as *T-cell receptor beta chain V region* and *major histocompatibility complex class II* (Supplementary Table 2).

## 4 Discussion

Currently, a major issue in the aquafeed industry, especially for salmonids, is the replacement of finite and limited FO in diet formulations with suitable alternative oils (Naylor et al., 2009). Although VO are presently being used as an alternative to FO, they are devoid of the n-3 LC-PUFA that, until now, were uniquely present only in FO. This practice translates into lower contents of EPA and DHA in fish tissues and consequently reduced intake to the consumers of farmed fish (Turchini et al., 2010, 2011). This problem is particularly pertinent in salmon because the same physiological characteristic, storage of oil in flesh, that makes salmon an excellent delivery system for n-3 LC-PUFA to the human consumer (Henriques et al., 2014), is also the characteristic that makes farmed salmon especially sensitive to changes in dietary fatty acid composition (Tocher, 2003). To solve this issue, a metabolically engineered terrestrial oilseed crop was specifically tailored to produce high contents of an n-3 LC-PUFA. Specifically, the transgenic *C. sativa* plants assessed in the present study yielded a seed oil (ECO) with 20% EPA (Betancor et al., 2015; Ruiz-Lopez et al., 2014), one of the key n-3 LC-PUFA and substrate for endogenous production of DHA, the other key n-3 LC-PUFA. Fish growth performance was not affected by the substitution of FO with either of the camelina oils, ECO and WCO, which was consistent with another study using regular camelina oil in salmon (Hixson et al., 2014). Indeed, the inclusion of ECO resulted in numerically the highest final weight indicating that it could be a suitable ingredient in feeds for on-growing Atlantic salmon with no detriment to performance and production.

The fatty acid composition of regular VOs, generally naturally rich in shorter chain fatty acids and PUFA, differs substantially from that of FO, and this can lead to differences in fatty acid digestibility with potential impact on absorption (Austreng et al., 1979; Sigurgisladottir et al., 1992). Additionally, it is not possible extrapolate from existing data on other VO, as the fatty acid profile of the transgenic-derived ECO is a hybrid between a VO and a FO. Therefore, it was necessary to determine the lipid and fatty acid digestibility of the ECO feed. Apparent digestibility coefficients (ADC) for lipid were generally high and were affected by dietary lipid source, but were actually slightly higher in the ECO and WCO feeds than in FO feed. This trend was also found in previous VO trials in Atlantic salmon (Menoyo et al., 2007) and Atlantic halibut (*Hippoglossus hippoglossus* L.) (Alves-Martins et al., 2009). In contrast, in temperate–warm water species such as European sea bass (Castro et al., 2014), red hybrid tilapia (*Oreochromis* spp.) (Ng et al., 2009) and Murray cod (*Maccullochella peelii peelii*) (Francis et al., 2007) the highest lipid ADC was observed in FO-fed fish. This could be due to physiological differences in lipid absorption mechanisms among fish species, as temperature does not generally greatly affect overall lipid ADC (Ng et al., 2004). Regarding the ADC for individual fatty acids, results were consistent with previous studies, where digestibility decreased with increasing chain length, but increased with increasing degree of unsaturation (Johnsen et al., 2000; Ng et al., 2004; Sigurgisladottir et al., 1992). This results in generally preferential absorption of PUFA followed by monoenes and finally saturated fatty acids. However, there were no negative effects of ECO in terms of fatty acid digestibility and, indeed, inclusion of the high-EPA oil led to intermediate individual fatty acid digestibilities between diets FO and WCO for most of the saturated fatty acids, 18:1n-9 or 20:2n-6 among others. Reflecting the higher lipid digestibility of the VO diets, most of the individual fatty acids actually showed slightly higher digestibility in the ECO and WCO feeds than in the FO feed. In particular, EPA in the ECO feed showed the numerically highest digestibility of all the feeds showing that the GM-derived EPA was, as expected, no different to FO-derived EPA. Thus, the transgenically produced EPA-enhanced VO can be included in salmon feeds with no negative impact on lipid and fatty acid digestibility, absorption and utilisation compared to FO or other VO.

Previously, we showed that the ECO diet increased EPA, but not DPA or DHA, in salmon flesh but, in liver, DPA and DHA were also significantly increased in addition to EPA (Betancor et al., 2015). This suggested that there was active conversion of EPA to DHA in the liver and increased expression of Δ6 and Δ5 fatty acyl desaturases and Elovl 5 and 2 in this tissue in fish fed ECO (and WCO) compared to fish fed FO was consistent with this. Therefore, in the present study we investigated the occurrence of this pathway in other relevant tissues including intestine, as this is known to have active LC-PUFA biosynthesis in salmonids (Bell et al., 2003; Fonseca-Madrigal et al., 2006; Tocher et al., 2004), and brain based on the importance of DHA in neural tissue. It was clear that there was conversion of dietary EPA to DPA and DHA in both anterior and, especially, pyloric caeca, in fish fed the ECO diet as shown by the higher levels of DPA and DHA compared to the levels in fish fed WCO. This activity in the pyloric caeca was confirmed by the gene expression data, where up-regulation of all the genes participating in LC-PUFA biosynthesis was observed. Thus, the high dietary EPA in the ECO feed did not suppress expression of the desaturases and elongases or resultant fatty acid desaturation and elongation, consistent with previous studies on the effects of EPA both in vitro (Thomassen et al., 2012) and in vivo (Betancor et al., 2015). It was noteworthy that the higher expression of the genes of LC-PUFA biosynthesis in intestine of fish both ECO and WCO compared to fish fed FO was also reflected in metabolism of dietary 18:3n-3 and 18:2n-6 as shown by higher levels of 20:3n-3 and 20:4n-3, and 18:3n-6, 20:2n-6, 20:3n-6 and 20:4n-6, respectively. These data were consistent with those from many studies that reported increased desaturation/elongation products after feeding VO in fish (Izquierdo et al., 2003; Menoyo et al., 2004; Morais et al., 2012a,b; Mourente et al., 2005).

In contrast, the fatty acid composition in brain was more conserved and less affected by diet, consistent with other studies in Atlantic salmon (Betancor et al., 2014; Brodtkorb et al., 1997) and other species (Benedito-Palos et al., 2010). Furthermore, although brain also showed the highest DHA content (20.9% on average), there were no differences among dietary treatments, probably reflecting selective deposition and/or a slow turnover of this fatty acid in neural tissue. In addition though, the data showed some elongation of dietary EPA with increased DPA in ECO-fed fish compared to both WCO- and FO-fed fish. Limited activation of the LC-PUFA biosynthesis from C_18_ PUFA was observed in brain with increased 20:3n-3, 20:4n-3 and 20:4n-6 as previously reported in this tissue (Betancor et al., 2014).

Extensive research has been performed on the effects of FO substitution with VO on tissue histopathology in several fish species (Bell et al., 2001; Caballero et al., 2004; Figuereido-Silva et al., 2005; Moldal et al., 2014; Mourente et al., 2007) and a common finding has been increased lipid deposition within hepatocytes (Aziza et al., 2013; Caballero et al., 2002, 2004; Kowalska et al., 2011). In the present study, the higher lipid vacuoles in hepatocytes observed histologically in fish fed both camelina oil diets was correlated with higher lipid content in this tissue, particularly in fish fed WCO (Betancor et al., 2015). Liver steatosis has been associated with deposition of VO-derived 18:1n-9 in this tissue (Caballero et al., 2002). However, in the present study, the percentage of 18:1n-9 in livers of fish fed ECO was half that of fish fed WCO (11.8% vs 22.0%), similar to the level in liver of fish fed FO (10.5%; Betancor et al., 2015). Therefore, it is possible that lipid deposition in salmon may be at least partly regulated by DHA levels, as this fatty acid was similarly low in both WCO and ECO feeds.

Melanomacrophage centres (MMC) are groupings of pigment-containing cells, mainly macrophages, within the stroma of the haematopoietic tissues spleen and the kidney. Changes in their size and/or distribution have been associated to environmental (Wolke et al., 1995), thermal (De Souza-Santos et al., 2014) and chronic stress (Agius and Roberts, 2003) as well as infectious diseases (Pronina et al., 2014). VO contain high levels of n-6 PUFA, which are considered pro-inflammatory based on the activity of the particular prostaglandins, leukotrienes and lipoxins derived from 20:4n-6 (Tocher, 2003), and can affect fish stress resistance, reduce pathogen resistance, and alter several immune system-related parameters (Montero and Izquierdo, 2010). Therefore, inclusion of WCO and ECO may alter fish stress homeostasis and lead to alterations in the size and distribution of the MMC. In this respect, a recent study in Nile tilapia demonstrated increased area occupied by MMC in liver when fish were fed diets containing VO (Aziza et al., 2013). In contrast to that previous study, no differences were found in the area of MMC in the head kidney of Atlantic salmon fed FO, WCO or ECO diets. Similarly, Atlantic salmon fed genetically-modified full fat soybean meal showed no differences in the number and size of MMC compared to fish fed FO (Sissener et al., 2009), so there could be species-related differences, as morphological differences in MMC between bony fish and cartilaginous fish have been reported (Agius and Roberts, 2003).

Although several studies on the effects of substitution of dietary FO by VO on tissue transcriptomes in teleosts have been performed (Limtipsuntorn et al., 2014; Morais et al., 2011a,b), relatively little is known about the effects on the fish intestinal tract and, in particular, pyloric caeca (Calduch-Giner et al., 2012; Morais et al., 2011a,b). The intestine is more than simply the site of nutrient uptake, and pyloric caeca have an active role in LC-PUFA biosynthesis as well as in digestion and absorption (Tocher et al., 2006; Vagner and Santigosa, 2011). The pyloric caeca transcriptome was affected differently by substitution of FO with ECO compared to the effects on liver transcriptome reported previously (Betancor et al., 2015). A higher number of DEG were found in liver compared to pyloric caeca and also the intensity of the response was generally higher in liver. This difference in the number and intensity of DEG found between these tissues is likely related to the functional roles of each organ. Liver is the major metabolic tissue in the body and thus is highly responsive to dietary change, leading to a larger transcriptional response in terms of genes expressed at different levels as well as the magnitude of the expression changes of these genes.

However, the cell processes and functional categories affected were generally similar in all the dietary comparisons for both tissues, although pathways included in the top 100 DEG varied between liver and pyloric caeca. A higher number of genes were regulated in pyloric caeca when fish fed ECO were compared to fish fed FO than when compared to fish fed WCO, in agreement with results obtained previously in liver (Betancor et al., 2015). Metabolism was the main category affected among these commonly expressed genes in pyloric caeca, which was also in agreement with the previous study in liver, however, amino acid metabolism was the most represented category in pyloric caeca, rather than lipid metabolism in the liver (Betancor et al., 2015). When the list was reduced to the top 100 genes affected, *aspartate aminotransferase* (*ast*), a gene belonging to amino acid metabolism, was the only one regulated in a different direction depending upon the comparison with fish fed ECO, down-regulated when compared to FO-fed fish, and up-regulated when compared to WCO-fed fish. A key enzyme of amino acid metabolism, *ast* catalyses the reversible transamination of the α-amino group from l-aspartate to α-ketoglutarate forming oxaloacetate and glutamate (Jeffery et al., 2000). Oxaloacetate participates in de novo synthesis of fatty acids as it is reduced in the cytosol to malate by malate dehydrogenase. Curiously this enzyme is also in the same list of the top 100 hits of commonly regulated genes between contrasts ECO vs FO and ECO vs WCO. These data may indicate effects on lipogenesis in fish fed ECO with down-regulation in *ast* perhaps suggesting lower lipogenesis compared to fish fed WCO, but up-regulation in *ast* suggesting higher lipogenesis compared to FO-fed fish. Thus, the high EPA level in the ECO feed partially inhibited the described lipogenesis/lipid deposition that occurs as a result of VO substitution (Morais et al., 2011a,b). However, with a dietary lipid content of 21% it is unlikely that fish fed any of the dietary treatments would have “high” lipogenic activity.

The main functional category affected in pyloric caeca of salmon after the substitution of dietary FO with VO was metabolism, which was not surprising bearing in mind the functions of this tissue. A higher representation of genes belonging to lipid, protein and carbohydrate metabolic pathways were found. This was in agreement with several studies analysing tissue transcriptomic responses after dietary FO substitution in fish in which lipid metabolism was a main category affected (Betancor et al., 2015; Leaver et al., 2008; Limtipsuntorn et al., 2014; Morais et al., 2011a,b). It was perhaps not surprising to find that two of the main pathways affected within the lipid metabolism category were LC-PUFA and steroid biosynthesis, which is consistent with previous studies (Leaver et al., 2008). Another DEG from the lipid metabolism category included in the top 100 was apolipoprotein A-IV (apoA-IV), which was up-regulated in pyloric caeca of fish fed ECO and WCO diets compared to fish fed FO. However, this gene was not altered in the liver of fish fed the same dietary treatments (Betancor et al., 2015), although the intestines of cod fed a diet with 66% of FO replaced by regular camelina oil also elicited up-regulation in this gene, which is secreted by enterocytes in response to lipid absorption (Green et al., 1980). It was previously suggested that increased apoA-IV may be related to changes in re-acylation mechanisms and phospholipid synthesis rates in response to dietary VO, resulting in the accumulation of lipid in fish tissues (Morais et al., 2012b). On the other hand, apoA-IV has been associated with reverse cholesterol transport, a process leading to the net movement of cholesterol from peripheral tissues back to the liver (Stan et al., 2003). In agreement, a high number of genes related to cholesterol synthesis were up-regulated in VO-fed fish, a common finding in salmon fed alternative sustainable feeds, likely in response to the lower cholesterol levels in VO diets (Betancor et al., 2015; Morais et al., 2011a,b).

In summary, the nutritionally enhanced VO, enriched in EPA derived from transgenic *C. sativa* assessed in the present study was demonstrated to be a potentially suitable substitute for FO in salmon feed. The high EPA oil possessed features of both FO and VO, which translated into a mixed response that was similar to FO and WCO in different aspects. Complete substitution of FO by ECO did not affect any of the growth performance parameters studied and enhanced apparent digestibility of individual fatty acids compared to FO. The ECO-fed fish maintained high levels of EPA in the tissues studied. In addition, pyloric caeca and intestine of fish fed the ECO diet displayed active conversion of dietary EPA to DHA resulting in higher tissue levels of DPA and DHA, and an activation of LC-PUFA biosynthesis from C_18_ PUFA. This result is consistent with the conclusion that suppression of expression of genes of LC-PUFA biosynthesis is due to DHA and that dietary EPA alone is not sufficient. Dietary ECO promoted slight lipid accumulation within hepatocytes, but lower than that observed in fish fed WCO. A small number of DEG could be attributed to the effect of ECO (311 features), with the most affected category being metabolism including several lipid metabolic pathways. Some of the effects of dietary ECO on salmon reflected its vegetable origin, and thus it will be of great interest to assess the effects of the next iteration of oil from genetically-modified *C. sativa*, that with a fatty acid signature more similar to that of FO (approx. 7% EPA and 7% DHA), which is currently being studied in salmon.

The following are the supplementary data related to this article.

Supplementary Table 1 Transcripts corresponding to the top 100 most significant annotated features exhibiting common differential expression in Atlantic salmon pyloric caeca fed ECO compared to fish fed either FO or WCO diets. Features are arranged by functional categories and within them by increasing p value (assessed by Welch t-test). The percentages of genes distribution is represented after removing features belonging to the same gene.

Supplementary Table 2 Transcripts corresponding to the top 100 most significant annotated features exhibiting differential expression in Atlantic salmon pyloric caeca fed FO compared to fish fed either WCO or ECO diets. Features are arranged by functional categories and within them by increasing p value (assessed by Welch t-test). The percentages of gene distribution is represented after removing features belonging to the same gene.

Supplementary data to this article can be found online at http://dx.doi.org/10.1016/j.aquaculture.2015.03.020.

## Figures and Tables

**Table 1:** Formulations, proximate and fatty acid compositions (percentage of fatty acids) of the experimental feeds.

	FO	WCO	ECO
*Feed ingredients (%)*			
Fish meal, NA LT 70	24.5	24.5	24.5
Fish meal, SA 68 Superprime	24.5	24.5	24.5
Soy protein concentrate (60%)	14.4	14.4	14.4
Wheat gluten	4.9	4.9	4.9
Wheat	12.8	12.8	12.8
Fish oil	17.5	–	–
Wild-type Camelina oil (Wt-CO)	–	17.5	–
EPA-Camelina oil (Tr-CO)	–	–	17.5
Monocalcium phosphate	0.6	0.6	0.6
Vitamins/minerals	0.8	0.8	0.8
Yttrium oxide	0.05	0.05	0.05

*Analysed composition*			
Dry matter (%)	91.8	91.3	92.6
Protein (%)	45.4	46.1	47.1
Fat (%)	24.9	23.1	23.9
Ash	8.6	8.5	8.5
Gross energy (Kj/g)	22.5	22.4	22.7

*Fatty acid composition (%)*			
Ʃ saturated^1^	30.7	13.2	17.4
Ʃ monounsaturated^2^	26.6	36.4	18.2
20:2n-6	0.2	1.2	1.2
20:3n-6	0.1	0.0	1.2
20:4n-6	0.9	0.1	2.5
Ʃ n-6 PUFA^3^	5.3	18.6	25.7
20:3n-3	0.1	0.8	1.0
20:4n-3	0.7	0.1	3.0
20:5n-3	15.9	2.1	18.7
22:5n-3	1.8	0.3	1.0
22:6n-3	11.2	2.5	2.4
Ʃ n-3 PUFA	33.2	31.2	38.0
Ʃ PUFA^4^	42.7	50.4	64.4
Total n-3 LC-PUFA	29.5	5.0	25.2

^1^Contains 15:0, 22:0 and 24:0. ^2^Contains 16:1n-9, 20:1n-11, 20:1n-7, 22:1n-9 and 24:1n-9. ^3^Contains 22:4n-6 and 22:5n-6;^4^contains C16 PUFA. Fish and FO, fish oil and respective feed. LC-PUFA, long-chain polyunsaturated fatty acid (sum of 20:4n-3, 20:5n-3 22:5n-3 and 22:6n-3). n.d.: not detected.

**Table 2:** Growth performance, survival, feed utilisation and basic biometry over the 7-week experimental period.

	FO	WCO	ECO
Final weight (g)	196.5 ± 26.3	200.5 ± 28.5	207.9 ± 26.5
Total length (cm)	25.0 ± 1.0	24.8 ± 1.4	25.0 ± 1.0
Survival (%)	100.0 ± 0.0	100.0 ± 0.0	100.0 ± 0.0
HSI	0.9 ± 0.1	0.9 ± 0.1	0.9 ± 0.1
VSI	9.5 ± 0.9	9.6 ± 1.2	9.9 ± 0.7
FI (g/tank)	4200.0 ± 155.0	4203.0 ± 97.0	4257.0 ± 216.0
FCR	0.9 ± 0.0	0.9 ± 0.0	0.9 ± 0.0
SGR	1.9 ± 0.0	1.9 ± 0.0	2.0 ± 0.1
k	1.3 ± 0.0	1.3 ± 0.0	1.3 ± 0.0

Data are means ± SD (n = 3). There were no significant differences between treatments in any parameter. FCR, feed conversion ratio; FI, feed intake; HSI, hepato-somatic index; k, condition factor; SGR, specific growth rate; VSI, viscera-somatic index.

**Table 3:** Apparent digestibility coefficient (ADC) of lipid and fatty acids in Atlantic salmon fed the three experimental diets differing in oil source.

	FO	WCO	ECO
Fat ADC	90.6 ± 1.3^b^	96.8 ± 0.1^a^	95.7 ± 0.3^a^
14:0	86.9 ± 1.8^b^	97.6 ± 0.2^a^	97.0 ± 0.0^a^
15:0	86.3 ± 0.5^c^	97.5 ± 0.2^a^	95.9 ± 0.2^b^
16:0	83.9 ± 0.9^c^	96.8 ± 0.2^a^	95.2 ± 0.3^b^
18:0	78.8 ± 1.4^c^	96.1 ± 0.4^a^	93.1 ± 0.7^b^
Total saturated^1^	84.3 ± 0.8^c^	96.5 ± 0.3^a^	94.1 ± 0.5^b^
16:1n-7	97.5 ± 0.3^b^	98.6 ± 0.1^a^	98.3 ± 0.1^a^
18:1n-9	96.5 ± 0.4^c^	99.2 ± 0.1^a^	98.3 ± 0.1^b^
18:1n-7	98.8 ± 0.0	98.0 ± 1.7	95.8 ± 0.9
20:1n-9	94.4 ± 0.6^c^	99.1 ± 0.1^a^	98.4 ± 0.2^b^
20:1n-7	88.5 ± 1.5^b^	99.2 ± 0.1^a^	98.4 ± 0.2^a^
22:1n-11	93.3 ± 0.8^c^	98.0 ± 0.1^a^	96.8 ± 0.1^b^
22:1n-9	93.2 ± 0.7^c^	98.7 ± 0.1^a^	97.4 ± 0.3^b^
Total monoenes^2^	95.9 ± 0.5^c^	99.0 ± 0.1^a^	98.1 ± 0.2^b^
18:2n-6	95.7 ± 0.4^b^	99.2 ± 0.0^a^	99.1 ± 0.1^a^
18:3n-6	88.5 ± 1.1^c^	96.4 ± 0.4^b^	99.7 ± 0.7^a^
20:2n-6	95.1 ± 0.6^c^	99.3 ± 0.1^a^	98.8 ± 0.1^b^
20:4n-6	98.8 ± 0.1^b^	97.4 ± 0.1^c^	99.6 ± 0.1^a^
Total n-6 PUFA^3^	96.0 ± 0.4^b^	99.2 ± 0.1^a^	99.2 ± 0.1^a^
18:3n-3	97.6 ± 0.2^b^	99.7 ± 0.0^a^	99.5 ± 0.1^a^
18:4n-3	99.3 ± 0.1	99.3 ± 0.0	99.6 ± 0.1
20:3n-3	92.0 ± 1.4^b^	99.4 ± 0.1^a^	99.2 ± 0.1^a^
20:4n-3	98.7 ± 0.2^b^	98.1 ± 0.1^c^	99.6 ± 0.1^a^
20:5n-3	99.3 ± 0.1^b^	98.5 ± 0.1^c^	99.6 ± 0.1^a^
22:5n-3	98.5 ± 0.2^a^	96.9 ± 0.1^b^	98.8 ± 0.2^a^
22:6n-3	98.1 ± 0.2^a^	96.6 ± 0.2^b^	96.7 ± 0.4^b^
Total n-3 PUFA	98.8 ± 0.1^b^	99.3 ± 0.0^a^	99.4 ± 0.1^a^
Total PUFA^4^	98.5 ± 0.1^b^	99.3 ± 0.1^a^	99.3 ± 0.1^a^

Data expressed as means ± SD (n = 3). Different superscript letters within a row denote significant differences among diets. Statistical differences were determined by one-way ANOVA with Tukey's comparison test (p < 0.05). ^1^Contains 15:0, 22:0 and 24:0. ^2^Contains 16:1n-9 and 24:1n-9. ^3^contains 22:4n-6 and 22:5n-6. ^4^Contains C16 PUFA. ECO, feed containing oil from transgenic Camelina. FO, fish oil feed. LC-PUFA, long-chain polyunsaturated fatty acids (sum of 20:4n-3, 20:5n-3, 22:5n-3 and 22:6n-3). WCO, feed containing oil from wild-type Camelina.

**Table 4:** Lipid content (percentage of wet weight) and fatty acid compositions (percentage of total fatty acids) of total lipid of pyloric caeca after 7 weeks of feeding the experimental diets.

	FO	WCO	ECO
Lipid content	28.5 ± 1.9	24.8 ± 1.9	24.3 ± 1.4
14:0	5.5 ± 0.1^a^	1.9 ± 0.1^b^	2.0 ± 0.1^b^
16:0	15.2 ± 0.1^a^	9.4 ± 0.2^c^	10.2 ± 0.3^b^
18:0	3.4 ± 0.0^b^	3.1 ± 0.0^c^	4.2 ± 0.1^a^
20:0	0.2 ± 0.0^c^	0.8 ± 0.0^b^	1.1 ± 0.0^a^
Total saturated^1^	24.7 ± 0.2^a^	15.5 ± 0.2^b^	18.0 ± 0.5^c^
16:1n-7	6.8 ± 0.1^a^	2.3 ± 0.1^b^	2.4 ± 0.0^b^
18:1n-9	18.4 ± 0.2^b^	21.8 ± 0.4^a^	16.1 ± 0.4^c^
18:1n-7	3.6 ± 0.0^a^	2.1 ± 0.0^c^	2.3 ± 0.1^b^
20:1n-11	0.3 ± 0.0	n.d.	n.d.
20:1n-9	3.1 ± 0.1^c^	9.4 ± 0.2^a^	5.4 ± 0.2^b^
20:1n-7	0.3 ± 0.0	0.3 ± 0.0	0.4 ± 0.0
22:1n-11	2.7 ± 0.3	2.4 ± 0.2	1.5 ± 1.2
22:1n-9	0.4 ± 0.0	1.3 ± 0.1	1.2 ± 1.0
Total monoenes^2^	36.3 ± 0.3^b^	40.3 ± 0.5^a^	30.0 ± 0.1^c^
18:2n-6	6.6 ± 0.1^c^	14.7 ± 0.3^b^	16.4 ± 0.0^a^
18:3n-6	0.2 ± 0.0^c^	0.3 ± 0.0^b^	0.8 ± 0.0^a^
20:2n-6	0.5 ± 0.0^c^	1.4 ± 0.1^b^	1.7 ± 0.0^a^
20:3n-6	0.3 ± 0.0^c^	0.4 ± 0.0^b^	1.2 ± 0.0^a^
20:4n-6	0.7 ± 0.1^c^	0.3 ± 0.0^b^	1.6 ± 0.0^a^
Total n-6 PUFA^3^	8.7 ± 0.1^c^	17.2 ± 0.3^b^	22.0 ± 0.1^a^
18:3n-3	2.0 ± 0.1	14.6 ± 0.6	7.5 ± 0.0
18:4n-3	1.7 ± 0.0^b^	2.1 ± 0.1^a^	1.3 ± 0.0^c^
20:3n-3	0.2 ± 0.0^b^	1.0 ± 0.0^a^	0.9 ± 0.0^a^
20:4n-3	1.1 ± 0.0^b^	1.0 ± 0.1^b^	2.3 ± 0.0^a^
20:5n-3	8.2 ± 0.1^b^	2.0 ± 0.0^c^	8.7 ± 0.1^a^
22:5n-3	3.0 ± 0.0^a^	0.8 ± 0.0^b^	2.8 ± 0.1^a^
22:6n-3	11.9 ± 0.4^a^	4.9 ± 0.3^c^	5.9 ± 0.2^b^
Total n-3 PUFA	28.1 ± 0.4^b^	26.4 ± 0.4^c^	29.5 ± 0.4^a^
Total PUFA^4^	39.0 ± 0.5^c^	44.2 ± 0.6^b^	52.1 ± 0.5^a^
EPA + DHA	20.1 ± 0.3^a^	6.9 ± 0.4^c^	14.7 ± 0.3^b^
EPA:DHA	0.7 ± 0.0^b^	0.4 ± 0.0^c^	1.5 ± 0.0^a^
n-3:n-6	3.2 ± 0.0^a^	1.5 ± 0.0^b^	1.3 ± 0.0^c^
EPA + DPA + DHA	23.1 ± 0.3^a^	7.7 ± 0.4^c^	17.5 ± 0.3^b^

Data expressed as means ± SD (n = 3). Different superscript letters within a row denote significant differences among diets. Statistical differences were determined by one-way ANOVA with Tukey's comparison test (p < 0.05). ^1^Contains 15:0, 22:0 and 24:0. ^2^Contains 16:1n-9 and 24:1n-9. ^3^Contains 22:4n-6 and 22:5n-6. ^4^Contains C16 PUFA. ECO, feed containing oil from transgenic Camelina. FO, fish oil feed; LC-PUFA, long-chain polyunsaturated fatty acids (sum of 20:4n-3, 20:5n-3, 22:5n-3 and 22:6n-3). WCO, feed containing oil from wild-type Camelina.

**Table 5:** Lipid content (percentage of wet weight) and fatty acid compositions (percentage of total fatty acids) of total lipid of anterior intestine after 7 weeks of feeding the experimental diets.

	FO	WCO	ECO
Lipid content	9.8 ± 3.2	9.2 ± 0.5	8.8 ± 3.2
14:0	4.4 ± 0.5^a^	2.3 ± 0.4^b^	2.1 ± 0.4^b^
16:0	15.9 ± 0.4^a^	10.8 ± 0.8^b^	12.5 ± 0.2^b^
18:0	4.0 ± 0.4	3.6 ± 0.6	5.2 ± 0.5
20:0	0.3 ± 0.1^b^	0.7 ± 0.0^a^	1.0 ± 0.0^a^
Total saturated^1^	25.2 ± 0.4^a^	17.8 ± 1.0^c^	21.3 ± 0.3^b^
16:1n-7	5.7 ± 0.6^a^	2.6 ± 0.4^b^	2.4 ± 0.4^b^
18:1n-9	17.4 ± 0.7^ab^	21.3 ± 1.9^a^	15.4 ± 1.5^b^
18:1n-7	3.4 ± 0.0^a^	1.9 ± 0.2^b^	2.3 ± 0.1^b^
20:1n-11	0.3 ± 0.1	0.0 ± 0.0	0.1 ± 0.2
20:1n-9	2.9 ± 0.3^c^	8.1 ± 0.3^a^	4.5 ± 0.1^b^
20:1n-7	0.3 ± 0.0	0.3 ± 0.0	0.3 ± 0.0
22:1n-11	2.5 ± 0.5	2.4 ± 0.3	2.2 ± 0.4
22:1n-9	0.3 ± 0.1	1.0 ± 0.0	0.5 ± 0.0
Total monoenes^2^	33.6 ± 1.9^ab^	38.8 ± 2.5^a^	28.5 ± 2.6^b^
18:2n-6	6.3 ± 0.3^b^	13.1 ± 0.7^a^	12.6 ± 0.1^a^
18:3n-6	0.2 ± 0.0	0.3 ± 0.0	0.5 ± 0.0
20:2n-6	0.5 ± 0.0^b^	1.3 ± 0.0^a^	1.5 ± 0.1^a^
20:3n-6	0.3 ± 0.0^b^	0.4 ± 0.1^b^	1.0 ± 0.1^a^
20:4n-6	1.2 ± 0.3^b^	0.6 ± 0.2^b^	2.3 ± 0.4^a^
Total n-6 PUFA^3^	8.9 ± 0.2^c^	15.9 ± 0.4^b^	18.2 ± 0.5^a^
18:3n-3	1.9 ± 0.1^c^	12.0 ± 0.6^a^	5.6 ± 0.3^b^
18:4n-3	1.4 ± 0.2^ab^	1.9 ± 0.2^a^	1.1 ± 0.0^b^
20:3n-3	0.2 ± 0.0^b^	0.8 ± 0.0^a^	0.7 ± 0.1^a^
20:4n-3	0.9 ± 0.1^b^	1.0 ± 0.0^b^	1.7 ± 0.1^a^
20:5n-3	7.5 ± 0.5^a^	2.6 ± 0.4^b^	8.1 ± 0.7^a^
22:5n-3	2.7 ± 0.2^a^	1.0 ± 0.1^b^	2.6 ± 0.2^a^
22:6n-3	16.0 ± 2.6^a^	8.1 ± 2.4^b^	11.6 ± 0.8^ab^
Total n-3 PUFA	30.6 ± 1.6	27.4 ± 2.2	31.4 ± 1.9
Total PUFA^4^	41.2 ± 1.5^b^	43.8 ± 1.7^b^	50.2 ± 2.3^a^
EPA + DHA	23.5 ± 2.1^a^	10.7 ± 2.7^b^	19.7 ± 1.5^a^
EPA:DHA	0.5 ± 0.1	0.3 ± 0.1	0.7 ± 0.0
n-3:n-6	3.4 ± 0.2	1.7 ± 0.2	1.7 ± 0.1
EPA + DPA + DHA	26.0 ± 1.9^a^	11.7 ± 2.8^b^	22.3 ± 1.6^a^

Data expressed as means ± SD (n = 3). Different superscript letters within a row denote significant differences among diets. Statistical differences were determined by one-way ANOVA with Tukey's comparison test (p < 0.05). ^1^Contains 15:0, 22:0 and 24:0. ^2^Contains 16:1n-9 and 24:1n-9. ^3^Contains 22:4n-6 and 22:5n-6; ^4^contains C16 PUFA. ECO, feed containing oil from transgenic Camelina. FO, fish oil feed; LC-PUFA, long-chain polyunsaturated fatty acids (sum of 20:4n-3, 20:5n-3, 22:5n-3 and 22:6n-3). WCO, feed containing oil from wild-type Camelina.

**Table 6:** Lipid content (percentage of wet weight) and fatty acid compositions (percentage of total fatty acids) of total lipid of brain after 7 weeks of feeding the experimental diets.

	FO	WCO	ECO
Lipid content	7.3 ± 0.1	8.4 ± 1.4	7.6 ± 0.8
14:0	1.1 ± 0.3	0.9 ± 0.3	0.7 ± 0.3
16:0	17.7 ± 0.4	15.1 ± 1.8	15.9 ± 1.0
18:0	7.6 ± 0.2	6.4 ± 1.2	7.2 ± 0.6
20:0	0.1 ± 0.0	0.4 ± 0.2	0.3 ± 0.1
Total saturated**^1^**	26.9 ± 0.5	23.2 ± 2.5	24.4 ± 1.3
16:1n-7	2.3 ± 0.3^a^	1.9 ± 0.3^ab^	1.7 ± 0.2^b^
18:1n-9	19.1 ± 0.8^ab^	20.5 ± 1.0^a^	18.4 ± 0.8^b^
18:1n-7	3.3 ± 0.2^a^	2.6 ± 0.1^b^	2.8 ± 0.1^b^
20:1n-11	0.1 ± 0.0	0.1 ± 0.1	0.1 ± 0.0
20:1n-9	1.6 ± 0.2^b^	3.8 ± 1.3^a^	2.2 ± 0.4^ab^
20:1n-7	0.3 ± 0.0	0.3 ± 0.0	0.3 ± 0.0
22:1n-11	0.3 ± 0.1	0.8 ± 0.5	0.6 ± 0.3
22:1n-9	0.3 ± 0.0^b^	0.6 ± 0.2^a^	0.4 ± 0.0^ab^
Total monoenes^2^	32.7 ± 1.4	35.0 ± 2.3	31.1 ± 1.5
18:2n-6	1.2 ± 0.3	5.1 ± 2.4	3.4 ± 1.4
18:3n-6	0.1 ± 0.0	0.1 ± 0.0	0.1 ± 0.1
20:2n-6	0.2 ± 0.0^b^	0.6 ± 0.2^a^	0.5 ± 0.1^a^
20:3n-6	0.1 ± 0.0^b^	0.3 ± 0.0^a^	0.4 ± 0.1^a^
20:4n-6	1.1 ± 0.0^b^	0.8 ± 0.2^c^	1.6 ± 0.0^a^
Total n-6 PUFA^3^	2.9 ± 0.3	7.0 ± 2.5	6.2 ± 1.6
18:3n-3	0.3 ± 0.1^b^	4.7 ± 2.3^a^	1.5 ± 0.6^ab^
18:4n-3	0.2 ± 0.1	0.7 ± 0.3	0.3 ± 0.1
20:3n-3	0.1 ± 0.0^c^	0.6 ± 0.1^a^	0.4 ± 0.0^b^
20:4n-3	0.3 ± 0.0^b^	0.6 ± 0.1^a^	0.7 ± 0.2^a^
20:5n-3	6.2 ± 0.2^a^	4.5 ± 0.6^b^	7.0 ± 0.1^a^
22:5n-3	2.2 ± 0.1^b^	1.6 ± 0.2^c^	3.0 ± 0.3^a^
22:6n-3	23.4 ± 1.9	18.4 ± 3.9	20.9 ± 2.6
Total n-3 PUFA	32.9 ± 1.9	31.0 ± 1.9	33.8 ± 1.9
Total PUFA^4^	35.9 ± 1.6	38.2 ± 0.8	40.1 ± 0.9
EPA + DHA	29.7 ± 2.0	22.8 ± 4.5	27.9 ± 2.5
EPA:DHA	0.3 ± 0.0	0.2 ± 0.0	0.3 ± 0.0
n-3:n-6	11.7 ± 1.8^a^	4.8 ± 1.7^b^	5.7 ± 1.6^b^
EPA + DPA + DHA	31.9 ± 2.0	24.5 ± 4.6	30.9 ± 2.8

Data expressed as means ± SD (n = 3). Different superscript letters within a row denote significant differences among diets. Statistical differences were determined by one-way ANOVA with Tukey's comparison test (p < 0.05). ^1^Contains 15:0, 22:0 and 24:0. ^2^Contains 16:1n-9 and 24:1n-9. ^3^Contains 22:4n-6 and 22:5n-6. ^4^Contains C16 PUFA. ECO, feed containing oil from transgenic Camelina. FO, fish oil feed; LC-PUFA, long-chain polyunsaturated fatty acids (sum of 20:4n-3, 20:5n-3, 22:5n-3 and 22:6n-3). WCO, feed containing oil from wild-type Camelina.

**Table 7:** Mean scores for the lipid vacuolization in liver and melanomacrophages area in head kidney of Atlantic salmon fed the experimental diets for seven weeks.

	FO	WCO	ECO
Cytoplasmic lipid vacuolization	0.2 ± 0.2^b^	1.7 ± 0.7^a^	1.5 ± 1.2^ab^
Melanomacrophage area (pixels)	7.9 × 10^9^ ± 3.3 × 10^8^	8.8 × 10^9^ ± 4.0 × 10^8^	7.3 × 10^9^ ± 3.7 × 10^8^

ECO, feed containing oil from transgenic Camelina; FO, fish oil feed; LC-PUFA, long-chain polyunsaturated fatty acids (sum of 20:4n-3, 20:5n-3, 22:5n-3 and 22:6n-3); WCO, feed containing oil from wild-type Camelina. Cytoplasmic lipid vacuolization score: 0, not observed; 1, few; 2, medium; 3, severe. Different superscript letters within a row denote significant differences among diets. Statistical differences were determined by one-way ANOVA with Tukey’'s comparison test (p < 0.05).

**Table 8:** Summary of the results of microarray analysis.

	ECO vs FO	ECO vs WCO	WCO vs FO
Total no of probes		44,000	
Total no of DEG	2298	1152	1404
Up-regulated genes	1367	661	865
FC 1–1.5	1031	476	647
FC 1.5–2.5	323	176	204
FC > 2.5	13	9	14
Down-regulated genes	931	491	539
FC 1–1.5	676	380	372
FC 1.5–2.5	237	98	156
FC > 2.5	18	13	11

ECO, feed containing oil from transgenic Camelina; FO, fish oil feed; LC-PUFA, long-chain polyunsaturated fatty acids (sum of 20:4n-3, 20:5n-3, 22:5n-3 and 22:6n-3); WCO, feed containing oil from wild-type Camelina.
