# Supplementary material for: Evaluation of a high-EPA oil from transgenic Camelina sativa in feeds for Atlantic salmon (Salmo salar L.): Effects on tissue fatty acid composition, histology and gene expression
Source: Aquaculture. 2015 Jul 1;444:1–12. doi: 10.1016/j.aquaculture.2015.03.020 (PMC4459488; doi:10.1016/j.aquaculture.2015.03.020)
Supplement: Supplementary Table 1 — Transcripts corresponding to the top 100 most significant annotated features exhibiting common differential expression in Atlantic salmon pyloric caeca fed ECO compared to fish fed either FO or WCO diets. Features are arranged by functional categories and within them by increasing p value (assessed by Welch t-test). The percentages of genes distribution is represented after removing features belonging to the same gene. [file mmc1.docx]

| **KO no**  Supplementary Table 1. Transcripts corresponding to the top 100 most significant annotated features exhibiting common differential expression in Atlantic salmon pyloric caeca fed ECO compared to fish fed either FO or WCO diets. Features are arranged by functional categories and within them by increasing p value (assessed by Welch t-test). The percentages of genes distribution is represented after removing features belonging to the same gene. | **ECO/FO** | | **ECO/WCO** | | **Annotation** | **Access no** |
| --- | --- | --- | --- | --- | --- | --- |
|  | **p** | **FC** | **p** | **FC** |  |  |
| *Metabolism (14.6 %)* | | | | | | |
| *Lipid (7.3 %)* | | | | | | |
| K16342 | 0.0004 | -1.34 | 0.0125 | -1.26 | Cytosolic phospholipase A2 | BX869790 |
| K00864 | 0.0005 | -1.71 | 0.0106 | -1.48 | Glycerol kinase | CA361471 |
| K00498 | 0.0029 | +1.23 | 0.0037 | +1.22 | Cholesterol monooxygenase | NP1611644 |
|  |  |  |  |  |  |  |
| *Carbohydrate (2.4 %)* | |  |  |  |  |  |
| K01196 | 0.0000 | +1.99 | 0.0323 | +1.57 | Glycogen debranching enzyme | S35505260 |
|  |  |  |  |  |  |  |
| *Amino acid (4.9 %)* | |  |  |  |  |  |
| K14455 | 0.0023 | -1.23 | 0.0473 | +1.21 | Aspartate aminotransferase | DY706451 |
| K00029 | 0.0035 | -1.60 | 0.0144 | -1.26 | Malate dehydrogenase | S35486251 |
|  |  |  |  |  |  |  |
| *Transport (4.9 %)* | |  |  |  |  |  |
| K12405 | 0.0006 | +1.11 | 0.0000 | +1.25 | 3-hydroxyacyl-CoA dehydrogenase | S35675613 |
|  | 0.0007 | +1.38 | 0.0437 | +1.19 | ATP-binding cassette | TC90747 |
|  |  |  |  |  |  |  |
| *Translation (17.1%)* | |  |  |  |  |  |
| K02947 | 0.0003 | +1.27 | 0.0270 | +1.13 | Small subunit ribosomal protein S10e | S35569834 |
| K14527 | 0.0014 | -1.40 | 0.0110 | -1.50 | Ribonuclease P | S30242788 |
| K03113 | 0.0035 | -1.25 | 0.0110 | -1.16 | Translation initiation factor 1 | S35493512 |
|  | 0.0039 | +1.16 | 0.0281 | +1.20 | Small subunit ribosomal protein S30e | TC89999 |
| K02917 | 0.0043 | +1.08 | 0.0364 | +1.06 | Large subunit ribosomal protein L35Ae | DR696464 |
| K02934 | 0.0043 | +14.43 | 0.0025 | +17.98 | Large subunit ribosomal protein L6e | S18885712 |
| K02893 | 0.0048 | +1.16 | 0.0197 | +1.17 | Large subunit ribosomal protein L23Ae | S35687976 |
|  |  |  |  |  |  |  |
| *Protein folding (4.9 %)* | |  |  |  |  |  |
|  | 0.0018 | -1.87 | 0.0079 | -1.58 | Vesicle transport protein SEC22 | TC89129 |
| K03873 | 0.0020 | +1.43 | 0.0007 | +1.51 | Transcription elongation factor B | CA346107 |
|  |  |  |  |  |  |  |
| *Signalling (17.1 %)* | |  |  |  |  |  |
| K13567 | 0.0000 | -1.61 | 0.0331 | -1.54 | Activin receptor type-1B | S34310833 |
| K07836 | 0.0007 | -1.48 | 0.0261 | -1.33 | Ras-related protein Rap-1B | S35544806 |
| K06058 | 0.0009 | +1.23 | 0.0339 | +1.19 | Deltex | S35560376 |
| K05854 | 0.0010 | +1.44 | 0.0375 | +1.30 | Tyrosine-protein kinase Lyn | S48410371 |
| K02677 | 0.0016 | +1.31 | 0.0005 | +1.13 | Classical protein kinase C | S35500409 |
| K17698 | 0.0020 | +1.32 | 0.0327 | +1.19 | FYN binding protein | CA058998 |
| K09558 | 0.0030 | -1.28 | 0.0005 | -1.38 | BCL2-associated athano gene 4 | S18099782 |
|  |  |  |  |  |  |  |
| *Digestive (7.3 %)* | |  |  |  |  |  |
| K06237 | 0.0010 | -1.45 | 0.0447 | -1.28 | Collagen, type IV, alpha | CA039113 |
| K07376 | 0.0022 | -1.61 | 0.0023 | -1.55 | Protein kinase, cGMP-dependent | TC105153 |
| K07371 | 0.0026 | +1.63 | 0.0251 | +1.37 | B-cell linker protein | S18891272 |
|  |  |  |  |  |  |  |
| *Immune system (19.5 %)* | |  |  |  |  |  |
| K10989 | 0.0000 | +2.03 | 0.0274 | +1.55 | Activation-induced cytidine deaminase | S35472109 |
| K04011 | 0.0000 | +1.22 | 0.0419 | +1.20 | Complement component (3b/4b) receptor 1 | S30239991 |
|  | 0.0002 | +1.59 | 0.0277 | +1.25 | SH3-domain binding protein 2 | TC74569 |
|  | 0.0002 | +1.37 | 0.0054 | +1.20 | Dedicator of cytokinesis protein 2 | TC74191 |
| K06450 | 0.0003 | +1.39 | 0.0371 | +1.22 | T-cell surface glycoprotein CD3 delta chain | S37438813 |
| K06453 | 0.0004 | +1.40 | 0.0069 | +1.26 | CD3Z antigen, zeta polypeptide | S37438816 |
| K06458 | 0.0020 | +1.69 | 0.0391 | +1.39 | CD8A antigen, alpha polypeptide | S43839059 |
| K08847 | 0.0029 | +1.47 | 0.0019 | +1.44 | Receptor-interacting serine/threonine-protein kinase 3 | S31995767 |
|  |  |  |  |  |  |  |
| *Miscellaneous or unknown function (14.6 %)* | | | |  |  |  |
|  | 0.0001 | +1.84 | 0.0040 | +1.84 | Zinc finger protein 576 | KSS4953 |
|  | 0.0002 | +1.66 | 0.0269 | +1.26 | Plastin 3 | S15275191 |
|  | 0.0033 | -1.50 | 0.0068 | -1.34 | Tetraspanin-3 | Ssa#STIR06508 |
|  | 0.0004 | +1.71 | 0.0237 | +1.28 | Tetraspanin-9 | S35665494 |
|  | 0.0028 | +1.78 | 0.0003 | +1.72 | Tropomodulin 4 | S35670869 |
|  | 0.0005 | -1.49 | 0.0012 | -1.51 | TBC1 domain family, member 16 | CX036256 |
