# Supplementary material for: Evaluation of a high-EPA oil from transgenic Camelina sativa in feeds for Atlantic salmon (Salmo salar L.): Effects on tissue fatty acid composition, histology and gene expression
Source: Aquaculture. 2015 Jul 1;444:1–12. doi: 10.1016/j.aquaculture.2015.03.020 (PMC4459488; doi:10.1016/j.aquaculture.2015.03.020)
Supplement: Supplementary Table 2 — Transcripts corresponding to the top 100 most significant annotated features exhibiting differential expression in Atlantic salmon pyloric caeca fed FO compared to fish fed either WCO or ECO diets. Features are arranged by functional categories and within them by increasing p value (assessed by Welch t-test). The percentages of gene distribution is represented after removing features belonging to the same gene. [file mmc2.docx]

Supplementary Table 2. Transcripts corresponding to the top 100 most significant annotated features exhibiting differential expression in Atlantic salmon pyloric caeca fed FO compared to fish fed either WCO or ECO diets. Features are arranged by functional categories and within them by increasing p value (assessed by Welch t-test). The percentages of genes distribution is represented after removing features belonging to the same gene.

| **KO no** | **FO/WCO** | | **FO/ECO** | | **Annotation** | **Access no** |
| --- | --- | --- | --- | --- | --- | --- |
|  | p | FC | p | FC |  |  |
| *Metabolism (39.6 %)* | | | | | | |
| *Lipid (22.0 %)* | | | | | | |
| K01640 | 0.0009 | +1.64 | 0.0273 | +1.31 | Hydroxymethylglutaryl-CoA lyase | BX889044 |
| K16342 | 0.0362 | -1.37 | 0.0143 | -1.48 | Cytosolic phospholipase A2 | S15294190 |
| K10205 | 0.0390 | +1.29 | 0.0007 | +1.55 | Elongation of very long chain fatty acids protein 2 | DW531881 |
| K00222 | 0.0494 | +1.52 | 0.0057 | +2.00 | Delta14-sterol reductase | DY694469 |
| K05917 | 0.0029 | +2.01 | 0.0054 | +1.89 | Sterol 14-demethylase | DY731118 |
| K05917 | 0.0003 | +1.76 | 0.0004 | +1.68 | Sterol 14-demethylase | DY731118_S |
| K00461 | 0.0472 | -1.23 | 0.0277 | -1.28 | Arachidonate 5-lipoxygenase | EG930234 |
| K14156 | 0.0389 | +1.41 | 0.0192 | +1.31 | Choline/ethanolamine kinase | KSS4003 |
| K10226 | 0.0415 | +1.44 | 0.0242 | +1.36 | Delta-6 desaturase | S18892244 |
| K00787 | 0.0211 | +1.47 | 0.0436 | +1.47 | Farnesyl diphosphate synthase | S30244374 |
| K00626 | 0.0189 | +1.51 | 0.0065 | +1.54 | Acetyl-CoA C-acetyltransferase | S30292033 |
| K00489 | 0.0320 | +2.08 | 0.0069 | +2.63 | Cholesterol 7alpha-monooxygenase | S31963464 |
| K00511 | 0.0179 | +1.90 | 0.0157 | +2.12 | Squalene monooxygenase | S31977705 |
| K15013 | 0.0106 | +1.51 | 0.0006 | +1.99 | Long-chain-fatty-acid-CoA ligase ACSBG | S35552995 |
| K01823 | 0.0220 | +1.80 | 0.0096 | +1.87 | Isopentenyl-diphosphate delta-isomerase | S35553677 |
| K05917 | 0.0281 | +1.74 | 0.0021 | +1.85 | Sterol 14-demethylase | DY741343 |
| K00722 | 0.0079 | +2.09 | 0.0389 | +1.68 | Globoside alpha-N-acetylgalactosaminyltransferase | S15295955 |
| K00710 | 0.0000 | +1.71 | 0.0002 | +1.57 | Polypeptide N-acetylgalactosaminyltransferase | DY731189 |
| K03766 | 0.0348 | +1.86 | 0.0194 | +1.75 | Beta-1,3-N-acetylglucosaminyltransferase 5 | S35455181 |
| K03847 | 0.0197 | -1.17 | 0.0080 | -1.22 | Alpha-1,6-mannosyltransferase | S35506259 |
| K08760 | 0.0007 | +4.07 | 0.0120 | +2.34 | Apolipoprotein A-IV | S31963680 |
| K14457 | 0.0078 | -3.62 | 0.0015 | -3.06 | 2-acylglycerol O-acyltransferase 2 | S35538062 |
|  |  |  |  |  |  |  |
| *Carbohydrate (3.3 %)* | | | | | | |
| K08080 | 0.0086 | +1.72 | 0.0304 | +1.72 | CMP-N-acetylneuraminate monooxygenase | S34421591 |
| K00036 | 0.0459 | +1.25 | 0.0053 | +1.29 | Glucose-6-phosphate 1-dehydrogenase | DW180117 |
| K01091 | 0.0088 | +1.90 | 0.0247 | +1.44 | Phosphoglycolate phosphatase | S31983783 |
|  |  |  |  |  |  |  |
| *Energy (2.2 %)* | | | | | | |
| K03940 | 0.0171 | +1.22 | 0.0040 | +1.18 | NADH dehydrogenase Fe-S protein 7 | CX248550 |
| K03941 | 0.0341 | +1.35 | 0.0106 | +1.48 | NADH dehydrogenase Fe-S protein 8 | KSS4421 |
|  |  |  |  |  |  |  |
| *Amino acid (9.9 %)* | | | | | | |
| K00463 | 0.0010 | -1.72 | 0.0257 | -1.44 | Indoleamine 2,3-dioxygenase | CU072774 |
| K01755 | 0.0060 | -1.16 | 0.0480 | -1.17 | Argininosuccinate lyase | S18150009 |
| K14455 | 0.0202 | +1.28 | 0.0474 | +1.20 | Aspartate aminotransferase, mitochondrial | S23936748 |
| K01425 | 0.0148 | +1.83 | 0.0254 | +1.74 | Glutaminase | TC159194 |
| K00657 | 0.0100 | +1.52 | 0.0073 | +1.59 | Diamine N-acetyltransferase | CL275Ctg1 |
| K14455 | 0.0016 | +1.49 | 0.0023 | +1.23 | Aspartate aminotransferase, mitochondrial | DY706451 |
| K02320 | 0.0234 | +1.16 | 0.0104 | +1.34 | DNA polymerase alpha subunit A | DY710741 |
| K00933 | 0.0229 | +1.67 | 0.0307 | +1.64 | Creatine kinase | DY725455 |
| K01755 | 0.0318 | -1.14 | 0.0264 | -1.17 | Argininosuccinate lyase | EG822252 |
| K00383 | 0.0376 | -1.52 | 0.0142 | -1.42 | Glutathione reductase | S31979815 |
| K05361 | 0.0254 | +1.17 | 0.0403 | +1.15 | Phospholipid-hydroperoxide glutathione peroxidase | S35516050 |
|  |  |  |  |  |  |  |
| *Nucleotide (2.2 %)* | | | | | | |
| K01509 | 0.0410 | -1.26 | 0.0286 | -1.43 | Adenosinetriphosphatase | S35550203 |
| K12324 | 0.0140 | -1.25 | 0.0424 | -1.27 | Atrial natriuretic peptide receptor B | S32008863 |
|  |  |  |  |  |  |  |
| *Transport (7.7 %)* | | | | | | |
| K05767 | 0.0204 | -1.47 | 0.0459 | -1.47 | Ras GTPase-activating-like protein IQGAP2/3 | BX317230 |
| K01365 | 0.0344 | -1.44 | 0.0114 | -1.61 | Cathepsin L | CX262263 |
| K08746 | 0.0012 | +1.22 | 0.0017 | +1.20 | Solute carrier family 27, member 2 | S15338889 |
| K10789 | 0.0041 | -2.70 | 0.0040 | -2.79 | Myeloperoxidase | CL175Ctg1 |
| K05642 | 0.0002 | -1.73 | 0.0078 | -1.46 | ATP-binding cassette, subfamily A (ABC1), member 2 | DW549449 |
| K05767 | 0.0277 | -1.51 | 0.0341 | -1.49 | Ras GTPase-activating-like protein IQGAP2/3 | S18885995 |
| K13883 | 0.0233 | -1.37 | 0.0332 | -1.32 | Rab-interacting lysosomal protein | S30287117 |
| K07374 | 0.0054 | +1.63 | 0.0176 | +1.51 | Tubulin alpha | S31964371 |
| K08746 | 0.0268 | +1.21 | 0.0294 | +1.24 | Solute carrier family 27, member 2 | AM402694 |
|  | | | | | | |
| *Transcription (4.4 %)* | | | | | | |
| K12873 | 0.0127 | +1.21 | 0.0199 | -1.09 | Bud site selection protein 31 | S34421816 |
| K12886 | 0.0007 | +1.87 | 0.0083 | +1.51 | Heterogeneous nuclear ribonucleoprotein K | KSS155 |
| K03144 | 0.0049 | +1.33 | 0.0491 | +1.32 | Transcription initiation factor TFIIH subunit 4 | KSS3942 |
| K12856 | 0.0045 | +1.44 | 0.0085 | +1.42 | Pre-mRNA-processing factor 8 | DW560686 |
|  |  |  |  |  |  |  |
| *Translation (7.7 %)* | | | | | | |
| K14284 | 0.0154 | -1.17 | 0.0034 | -1.25 | Nuclear RNA export factor 1/2 | S18532972 |
| K02898 | 0.0386 | -1.06 | 0.0092 | -1.09 | Large subunit ribosomal protein L26e | KSS1822 |
| K14299 | 0.0018 | -1.69 | 0.0088 | -1.48 | Nucleoporin SEH1 | KSSb2269 |
| K14328 | 0.0127 | -1.18 | 0.0162 | -1.23 | Regulator of nonsense transcripts 3 | S30243750 |
| K01867 | 0.0008 | -1.12 | 0.0473 | +1.39 | Tryptophanyl-tRNA synthetase | S30246283 |
| K03239 | 0.0225 | +1.56 | 0.0267 | +1.36 | Translation initiation factor eIF-2B subunit alpha | S30276225 |
| K02921 | 0.0036 | -1.14 | 0.0250 | -1.21 | Large subunit ribosomal protein L37Ae | S35515995 |
|  |  |  |  |  |  |  |
| *Protein folding (4.4 %)* | | | | | | |
| K14012 | 0.0124 | +1.33 | 0.0274 | +1.34 | UBX domain-containing protein 1 | CA044316 |
| K10609 | 0.0222 | +1.29 | 0.0087 | +1.20 | Cullin 4 | CA062306 |
| K14012 | 0.0447 | +1.39 | 0.0150 | +1.43 | UBX domain-containing protein 1 | EG856480 |
| K09540 | 0.0151 | -1.40 | 0.0074 | -1.39 | Translocation protein SEC63 | S30276069 |
| K10610 | 0.0017 | +1.26 | 0.0007 | +1.32 | DNA damage-binding protein 1 | S30294495 |
|  |  |  |  |  |  |  |
| *Signalling (20.9 %)* | | | | | | |
| K03096 | 0.0485 | -1.22 | 0.0462 | -1.21 | Frequently rearranged in advanced T-cell lymphomas 2 | BX302182 |
| K05096 | 0.0264 | -1.36 | 0.0366 | -1.48 | FMS-like tyrosine kinase 1 | CX720066 |
| K05070 | 0.0218 | -1.27 | 0.0054 | -1.29 | Interleukin 2 receptor gamma | gi185133681 |
| K04273 | 0.0488 | +1.39 | 0.0002 | +1.62 | Lysophosphatidic acid receptor 6 | S15327334 |
| K05264 | 0.0016 | +1.79 | 0.0006 | +1.56 | Vasoactive intestinal peptide | S19712960 |
| K06236 | 0.0002 | -2.16 | 0.0147 | -1.41 | Collagen, type I/II/III/V/XI/XXIV/XXVII, alpha | S23930476 |
| K04137 | 0.0155 | +1.77 | 0.0048 | +1.74 | Adrenergic receptor alpha-1D | TC162380 |
| K04659 | 0.0057 | -1.65 | 0.0217 | -1.46 | Thrombospondin 2/3/4/5 | CB511693 |
| K04189 | 0.0476 | -1.40 | 0.0062 | -1.53 | C-X-C chemokine receptor type 4 | CL65Contig1 |
| K02187 | 0.0373 | -1.17 | 0.0059 | -1.25 | Caspase 3 | gi223649329 |
| K01312 | 0.0039 | -2.10 | 0.0064 | -2.56 | Trypsin | S18848194 |
| K05237 | 0.0301 | -1.93 | 0.0001 | -1.98 | Somatostatin | S18868047 |
| K15637 | 0.0428 | +1.27 | 0.0088 | +1.40 | Serine/threonine-protein phosphatase PGAM5 | S30240591 |
| K04674 | 0.0253 | +1.46 | 0.0247 | +1.47 | TGF-beta receptor type-1 | S30241522 |
| K11584 | 0.0346 | +1.25 | 0.0094 | +1.36 | Serine/threonine-protein phosphatase 2A regulatory subunit B' | S30294636 |
| K11220 | 0.0034 | +1.43 | 0.0226 | +1.41 | Signal transducer and activator of transcription 1 | S31981477 |
| K17693 | 0.0007 | -1.50 | 0.0029 | -1.56 | DNA-binding protein inhibitor ID2 | S35534464 |
| K04402 | 0.0125 | -1.60 | 0.0236 | -1.72 | Growth arrest and DNA-damage-inducible protein | S35553806 |
| K10867 | 0.0085 | -1.13 | 0.0078 | -1.14 | Nijmegen breakage syndrome protein 1 | S35529265 |
|  |  |  |  |  |  |  |
| *Digestive system (3.3 %)* | | | | | | |
| K05031 | 0.0250 | -1.70 | 0.0205 | -1.63 | Cystic fibrosis transmembrane conductance regulator | S18892351 |
| K05011 | 0.0189 | -2.14 | 0.0411 | -1.84 | Chloride channel 2 | S31972144 |
| K14208 | 0.0239 | +1.15 | 0.0062 | +1.14 | Xaa-Pro aminopeptidase 2 | S32004877 |
|  |  |  |  |  |  |  |
| *Immune system (6.6 %)* | | | | | | |
| K06856 | 0.0086 | -1.44 | 0.0023 | -1.88 | Immunoglobulin heavy chain | gi58201859 |
| K08893 | 0.0007 | -1.61 | 0.0195 | -1.39 | Hemopoietic cell kinase | CL213Ctg1 |
| K10785 | 0.0211 | -1.02 | 0.0013 | -1.00 | T-cell receptor beta chain V region | CL458Ctg1_S |
| K01334 | 0.0004 | -2.19 | 0.0205 | -2.33 | Component factor D | S18848091 |
| K06752 | 0.0013 | -1.94 | 0.0082 | -1.73 | Major histocompatibility complex, class II | S18892324 |
| K10785 | 0.0396 | -1.24 | 0.0132 | -1.30 | T-cell receptor beta chain V region | S18892491 |
| K06752 | 0.0081 | -1.59 | 0.0199 | -1.54 | Major histocompatibility complex, class II | S18892497 |
| K08011 | 0.0268 | +1.76 | 0.0244 | +1.75 | Neutrophil cytosolic factor 1 | S25806764 |
|  |  |  |  |  |  |  |
| *Miscellaneous and other categories (5.5 %)* | | | | | | |
| K00515 | 0.0209 | -1.82 | 0.0330 | -1.60 | Beta-carotene 15,15'-monooxygenase | S35496709 |
| K08008 | 0.0119 | -1.78 | 0.0237 | -1.83 | NADPH oxidase | CL177Ctg1 |
| K06572 | 0.0170 | -1.58 | 0.0043 | -1.45 | Plexin C | S35559285 |
| K15290 | 0.0231 | +1.38 | 0.0074 | +1.51 | Synaptotagmin-1 | CB499337 |
| K12327 | 0.0480 | -1.30 | 0.0060 | -1.39 | Caldesmon | S18892078 |
